# Novel Co-Occurrence of Germline *EGFR* p.V843I and Somatic *EGFR* Exon 19 Deletion in NSCLC: Insights into Reduced Sensitivity to EGFR-TKIs

**DOI:** 10.3390/ijms27167260

**Published:** 2026-08-14

**Authors:** Katarina Resen, Linea Cecilie Melchior, Jens Benn Sørensen, Karin Anna Wallentin Wadt, Edyta Maria Urbanska, Eric Santoni-Rugiu

**Affiliations:** 1 Department of Pathology, Rigshospitalet, Copenhagen University Hospital, 2100 Copenhagen, Denmark; katarina.resen.andersen@regionh.dk (K.R.); linea.cecilie.melchior@regionh.dk (L.C.M.); 2 Department of Oncology, Rigshospitalet, Copenhagen University Hospital, 2100 Copenhagen, Denmark; jens.benn.soerensen@regionh.dk (J.B.S.); edyta.maria.urbanska@regionh.dk (E.M.U.); 3 Department of Clinical Medicine, University of Copenhagen, 2200 Copenhagen, Denmark; karin.wadt@regionh.dk; 4 Department of Clinical Genetics, Rigshospitalet, Copenhagen University Hospital, 2100 Copenhagen, Denmark

**Keywords:** germline *EGFR* variants, p.V843I variant, EGFR-TKI intrinsic resistance, exon 19del, NSCLC

## Abstract

Germline *EGFR* pathogenic variants (PVs) are rare and define a distinct hereditary subset of NSCLC with unique clinical characteristics. Germline *EGFR* p.T790M is the most frequent and best characterized, with reported sensitivity to first- and second-generation EGFR-TKIs. Yet, the response of germline *EGFR* variants, especially the rarer ones such as p.V843I, to osimertinib remains poorly characterized. Given the very low frequency of germline non-p.T790M variants, their clinical relevance can only be investigated via case reports. Herein, we report what is, to the best of our knowledge, the first case of advanced lung adenocarcinoma harboring a germline *EGFR* p.V843I variant coexisting *in cis* with the unusual somatic *EGFR* exon 19 C-helix deletion, p.S752_I759del. Additionally, a somatic *TP53* variant was detected. Treatment with afatinib induced a partial response lasting only six months, as rapid disease progression occurred without identifiable acquired resistance mechanisms. Subsequently, no objective response to osimertinib or afatinib rechallenge was observed. Acquired *EGFR* and *MET* amplification were detected in corresponding rebiopsies. Overall survival was 29 months. Our findings suggest that, despite the presence of the previously reported EGFR-TKI-sensitive, *EGFR* p.S752_I759del, the co-occurrence of germline p.V843I may have contributed to reduced sensitivity to afatinib and osimertinib. This case expands the molecular spectrum of hereditary *EGFR*-mutated NSCLC and, together with our narrative review of the literature, supports an emerging model in which germline *EGFR* PVs may act both as tumor-predisposing events and as potential mechanisms of early resistance to EGFR-TKIs.

## 1. Introduction

Somatic *EGFR* variants are established oncogenic drivers and predictive biomarkers for EGFR-targeted therapy in non-small-cell lung cancer (NSCLC). In contrast, the biological and clinical significance of germline *EGFR* variants remains incompletely characterized [1]. The increasing implementation of next-generation sequencing (NGS) has led to the identification of a growing number of hereditary *EGFR* alterations; yet, their roles in NSCLC predisposition and treatment response are still unclear [1]. Germline *EGFR* variants are rare and mostly detected in advanced-stage NSCLC in never-smokers, although their penetrance and genotype–phenotype correlations are not completely characterized [1,2,3].

Germline *EGFR* pathogenic variants (PVs) account for less than 0.1% of *EGFR* alterations identified in patients with NSCLC, with p.T790M being the most frequently reported worldwide [1,4]. In contrast, somatic activating *EGFR* variants occur in approximately 10–15% of Caucasian patients with NSCLC and in up to 75% of East Asian patients with lung adenocarcinoma (LUAD), particularly among never-smokers, females, and younger patients [5]. Several germline *EGFR* PVs have been associated with familial clustering of LUAD and may not only predispose to lung carcinogenesis but also influence the biological behavior and therapeutic vulnerability of *EGFR*-driven tumors [2,6,7,8,9,10,11].

Recent evidence suggests that germline *EGFR* PVs frequently coexist with secondary somatic *EGFR* alterations that contribute to malignant transformation and progression. In the prospective INHERIT EGFR study, approximately 95% of lung cancers arising in germline *EGFR* carriers harbored additional somatic *EGFR* driver variants, supporting a model of cooperative oncogenic evolution in hereditary *EGFR*-associated NSCLC [12,13,14,15,16]. However, some germline *EGFR* PVs have been reported in the absence of a concomitant second driver alteration, raising the possibility that certain hereditary *EGFR* variants possess sufficient oncogenic potential to contribute directly to tumorigenesis. Nevertheless, undetected cooperating genomic events cannot be excluded given the limitations of current molecular profiling approaches [14,16,17]. The spectrum of germline *EGFR* PVs is heterogeneous and appears to differ across ethnic populations [1,17,18,19,20]. In Caucasians, p.T790M is the most frequently reported germline *EGFR* variant, occurring in 0.5–1% of never-smokers with NSCLC [8,9,10,11]. In contrast, the germline p.V843I in exon 21 is considerably less frequent, and its oncogenic and clinical significance is incompletely understood [21,22,23,24,25,26]. Several additional germline *EGFR* PVs have been described, highlighting the marked genetic heterogeneity of hereditary *EGFR*-driven NSCLC and potential ethnic differences in variant distribution [1,17,18,19,20,26]. Most reported variants affect the tyrosine kinase domain (TKD) and predominantly occur in female never-smokers with LUAD, although rare cases have also been described in squamous cell carcinoma and other NSCLC subtypes [17,18,19,20,26].

Among germline *EGFR* PVs, p.T790M and p.V843I are of particular interest because both have been associated with familial lung cancer and altered EGFR-TKI sensitivity. The germline p.T790M PV itself may behave as a relatively indolent oncogenic event, whereas coexistence with a secondary somatic *EGFR* variant, typically an exon 19 deletion (ex19del) or p.L858R, appears to confer a substantially stronger oncogenic effect [2,6,12,13,14,15,16]. Similarly, germline p.V843I has been reported in familial NSCLC and has been associated with variable sensitivity to EGFR-targeted therapies [21,22,23,24,25,26]. Structurally, the p.V843I substitution is located within the TKD of *EGFR* and has been hypothesized to alter the conformation of the ATP-binding pocket, potentially reducing the binding affinity of first-generation EGFR-TKIs in a manner analogous to the better-characterized p.T790M resistance variant [23,24]. These observations suggest that some germline *EGFR* PVs may influence sensitivity to EGFR-targeted therapies and contribute to intrinsic resistance, which is biologically distinct from the acquired resistance mechanisms that emerge under treatment pressure [23,27].

Despite increasing recognition of germline *EGFR* variants, the evidence regarding their sensitivity to second- and third-generation EGFR-TKIs is heterogeneous and largely depends on the specific germline variant. While recent data indicate that patients with germline *EGFR* p.T790M derive clinical benefit from osimertinib comparable to that observed in tumors harboring somatic p.T790M [2], evidence for less common germline variants, including p.V843I, remains limited to isolated case reports and small retrospective series [25,27,28,29]. Furthermore, the mechanisms underlying intrinsic resistance in this setting are insufficiently understood and may differ from the canonical acquired resistance pathways observed in sporadic *EGFR*-mutated NSCLC [30,31].

Given the rarity of germline *EGFR*-associated NSCLC, the biological behavior, therapeutic sensitivity, and resistance mechanisms of individual germline *EGFR* variants are still poorly elucidated [1,2,3,20]. Here, we report what is, to our knowledge, the first case of advanced LUAD harboring the germline *EGFR* p.V843I co-occurring *in cis* with a rare *EGFR* ex19del, the C-helix deletion p.S752_I759del. This unique molecular configuration provided an opportunity to further explore the interaction between hereditary and acquired *EGFR* alterations, molecular evolution, and reduced EGFR-TKI sensitivity. We therefore present this novel case in the context of a narrative review of the literature on germline *EGFR* PVs. In this way, we provide additional insight into hereditary *EGFR*-mutant NSCLC and discuss the complex role that *EGFR* germline–somatic variant interactions play in shaping tumor evolution and therapeutic resistance [15,16].

## 2. Results

**Figure 1:**
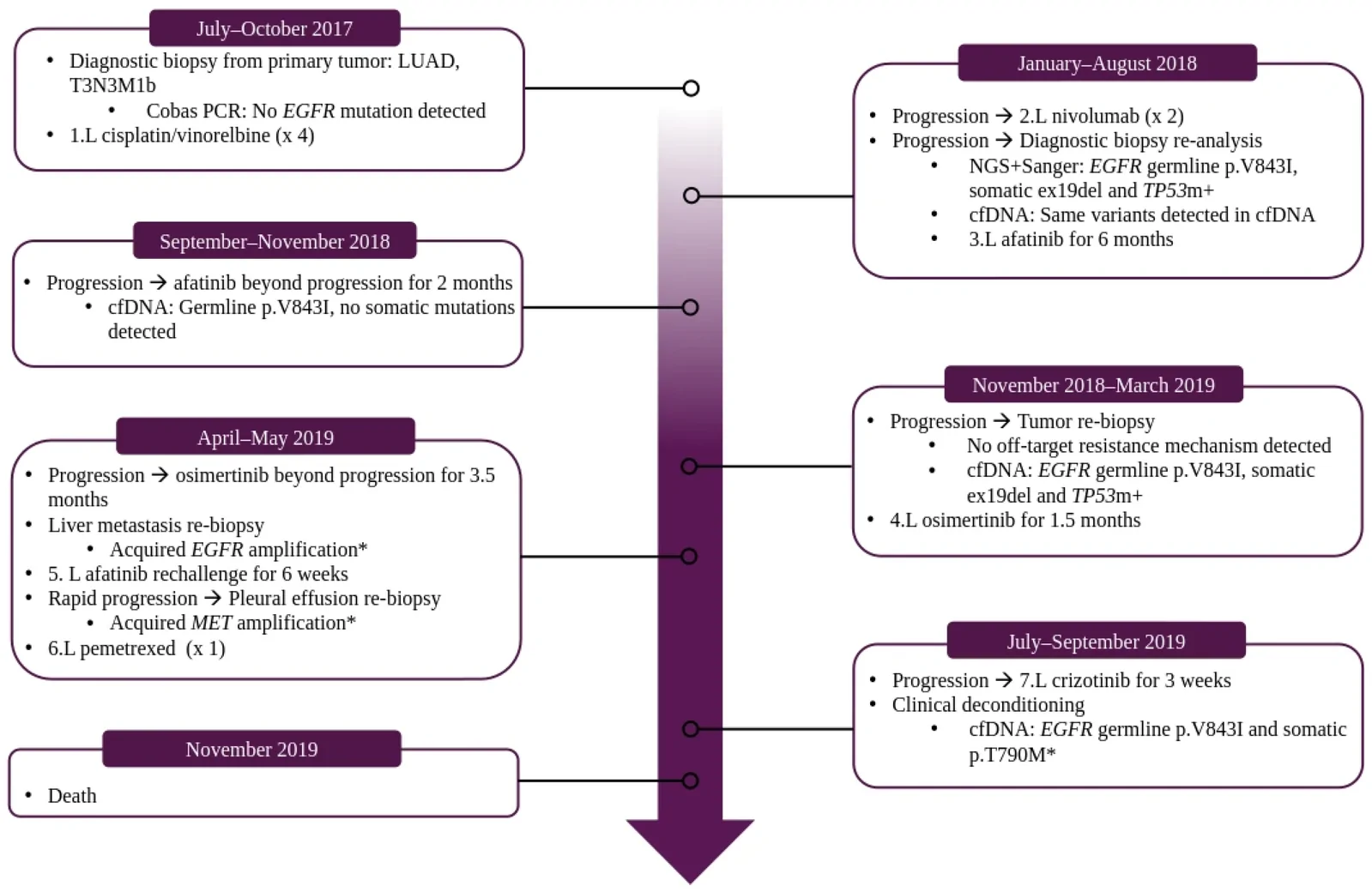
Tumor and treatment journey. Abbreviations: 1–7.L: first-, second-, third-, fourth-, fifth-, sixth- and seventh-line therapy. m+: mutated. * In addition to the original mutational profile (germline *EGFR* p.V843I, somatic *EGFR* ex19del and *TP53*m+).

The proband was a 44-year-old female, never-smoker, without comorbidities, diagnosed in July 2017 with LUAD in the right upper lobe with spread to mediastinal and cervical lymph nodes, malignant pericardial effusion, and multiple skeletal metastases (T3N3M1b; TNM 8th edition). At that time, the diagnostic transthoracic biopsy of the LUAD was examined by immunohistochemistry (IHC), which showed no expression of ALK, ROS1 or PD-L1 proteins, while a real-time PCR assay (Cobas^®^ *EGFR* Mutation Test v2, Roche Diagnostics) did not reveal any *EGFR* variants. Accordingly, the patient received four cycles of cisplatin/vinorelbine chemotherapy (Figure 1). The following CT scan showed stable disease, but the patient experienced considerable toxicity and did not accept pemetrexed maintenance treatment. CT scan after two months revealed radiological progression and immunotherapy with the PD-1 inhibitor nivolumab was initiated, as the patient still declined further chemotherapy. CT scan after two cycles showed further systemic progression, including new multiple choroidal metastases. Thus, given the young age and never-smoking status, the initial diagnostic tumor biopsy was reanalyzed in February 2018 by targeted NGS (Ampliseq Colon-Lung Cancer Panel v2, ThermoFisher Scientific), which revealed two variants that were not detected in the parallel NGS analysis of the patient’s blood lymphocytes, hence confirming that they were somatic. One variant was the *EGFR* ex19del, p.S752_I759del that belongs to the less frequent category of ex19dels known as “C-helix” deletions [32,33]. The other one was the *TP53* missense variant p.E258K. Both somatic variants were considered pathogenic in the Catalogue Of Somatic Mutations In Cancer (COSMIC) [34]. As with other C-helix deletion variants, the p.S752_I759del is *EGFR*-activating and exhibits similar patterns of TKI sensitivity as other *EGFR* ex19dels, but it results from a larger deletion of 24 nucleotides (24n-del) in exon 19 (c.2254_2277del24), eliminating eight amino acids, from S752 to I759, in the TKD [32,33]. The p.S752_I759del was not identified by the Cobas real-time PCR, as the latter does not cover this infrequent ex19del.

**Figure 2:**
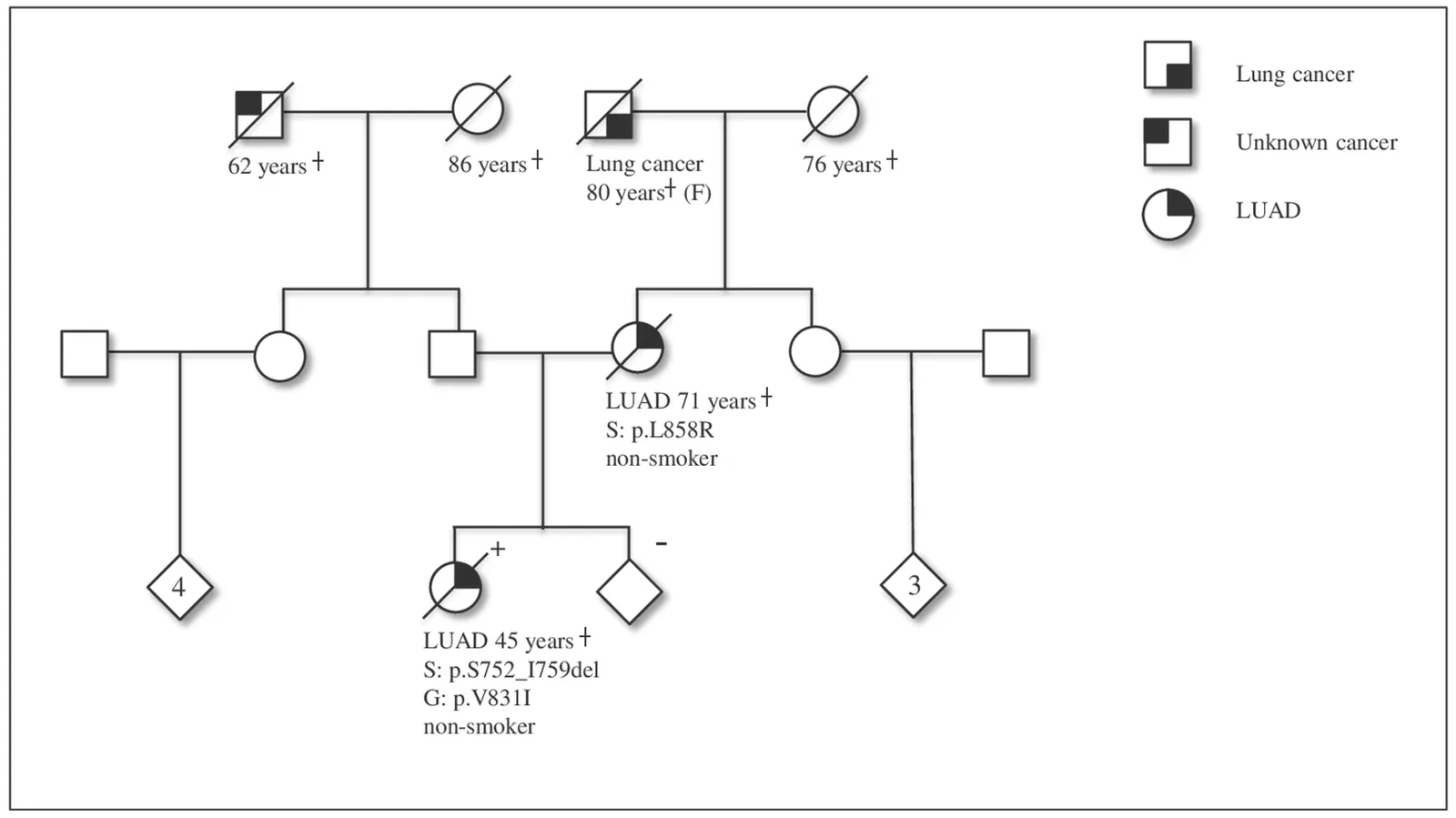
Pedigree chart of the patient’s family. Squares indicate males, circles females, and diamonds unspecified individuals or grouped offspring. Numbers within diamonds indicate the number of individuals in that branch. Slashed symbols and the dagger (†) indicate deceased family members. The plus (+) symbol indicates confirmed presence of germline *EGFR* p.V843I variant. The minus (−) symbol indicates no presence of germline *EGFR* p.V843I variant. Abbreviations: LUAD: lung adenocarcinoma; S: somatic variant; G: germline variant.

Given the family history of LUAD, the patient was referred to genetic counseling. Since we suspected a possible inheritable predisposition, Sanger sequencing of the *EGFR* gene was performed on DNA from circulating lymphocytes and from diagnostic biopsy, given that not all possible variants in *EGFR* were covered by our initial targeted NGS panel. In both samples, we identified the pathogenic germline *EGFR* c.2527G>A, p.V843I variant in exon 21, which was localized *in cis* to the somatic ex19del found in the LUAD. Simultaneous analysis of circulating free (cf)DNA from plasma was performed using a combination of NGS and Sanger sequencing and displayed the same two somatic *EGFR* and *TP53* variants, together with the germline p.V843I variant, as found in the primary tumor. Accordingly, the patient initiated third-line treatment with afatinib 30 mg once daily (QD) in March 2018, resulting in rapid clinical improvement and good tolerability, enabling a return to work. After one month of treatment, a ^18^FDG-PET/CT scan revealed a partial response, according to RECIST1.1 criteria. Concomitantly tested cfDNA was without the somatic *EGFR* ex19del and *TP53* variants but maintained the germline p.V843I variant. The lack of *EGFR* ex19del and maintenance of p.V843I in the cfDNA was further verified by an additional NGS assay (Oncomine Focus Assay, ThermoFisher Scientific) that is more sensitive and has broader coverage of *EGFR* variants than the initial Ampliseq NGS panel. These results suggested that the *EGFR*-dependent LUAD cells carrying the combined germline and somatic variants might have been temporarily eliminated or their allele frequency significantly reduced under the limit of detection of the assays, so that the germline p.V843I detected in cfDNA may have been primarily, if not entirely, derived from blood cells. The deceased mother of the proband had previously been diagnosed at 71 years of age with metastatic LUAD harboring a somatic *EGFR* p.L858R variant and received the EGFR-TKI, erlotinib, for 15 months before succumbing to the disease (Figure 2). Resequencing of the limited formalin-fixed, paraffin-embedded (FFPE) residual tumor tissue was inconclusive owing to insufficient DNA content. Thus, we could not confirm the occurrence of the germline *EGFR* p.V843I pathogenic variant in the deceased mother of the proband, although this was very likely. In addition, the family reported lung cancer in the maternal grandfather at age 80, consistent with maternal transmission of the variant, although the grandfather’s genetic status was not known. Pre-symptomatic genetic testing was offered to family members; however, no additional carriers were identified (Figure 2).

After 6 months of afatinib treatment, ^18^FDG-PET/CT showed progression with new liver metastases, while the choroidal metastases were in regression. As the patient’s performance status remained stable, treatment was continued beyond radiographic progression for an additional two months. Analysis of cfDNA at this time revealed re-appearance of the *EGFR* ex19del and persistence of the germline *EGFR* p.V843I variant. Consistent with these findings, rebiopsy from the progressing lung tumor still showed LUAD with these two *EGFR* variants and without phenotypical mechanisms of resistance to EGFR-TKIs, such as transformation to small cell carcinoma, squamous cell carcinoma, or epithelial–mesenchymal transition [31]. NGS on DNA (Oncomine Focus Assay, ThermoFisher Scientific) and RNA (Archer^®^ FusionPlex Solid Tumor panel; Archer^®^) isolated from the biopsy did not detect any gene variant, amplification or fusion that could represent a mechanism of resistance to afatinib and tumor progression. Given the similarity of p.V843I to p.T790M [23,24,28], the patient was offered next-line treatment with osimertinib. She wished to start with half the dose, but after moderate progression observed by ^18^FDG-PET/CT after 6 weeks of treatment, the dose was increased to standard 80 mg QD. A new biopsy from progressing liver metastases showed persistence of the uncommon *EGFR* ex19del and germline p.V843I variant as well as an acquired *EGFR* amplification (eight copies).

After further radiological progression 2 months later, a re-challenge with afatinib was attempted; however, there was no response (Figure 1). The patient’s condition rapidly deteriorated, with increasing dyspnea due to pleural effusion. Thoracocentesis provided relief to the patient and showed metastatic LUAD cells with the known *EGFR* ex19del and germline p.V843I variant. However, no on-target osimertinib-resistant variants such as p.C797S or signs of phenotypical transformation were detected in this sample. Instead, both NGS and fluorescence in situ hybridization (FISH) as well as IHC demonstrated the presence of metastatic cells with high-level *MET* amplification (average number of *MET* copies/tumor cell nucleus = 8; ratio *MET*/CEN7 = 3; >60% of tumor cell nuclei with ≥6 *MET* copies; 15% of tumor cell nuclei with “*MET* clusters” corresponding to ≥15 copies) and MET overexpression (MET IHC 3+ in 100% of tumor cells) [35]. Accordingly, the patient received crizotinib at a standard dose; however, after only 3 weeks, new clinical progression occurred. Analysis of plasma cfDNA at this stage revealed a newly acquired somatic *EGFR* p.T790M variant, in addition to the known germline p.V843I variant, suggesting the occurrence of a new p.T790M—harboring cancer clone, possibly selected during treatment and/or re-challenge with afatinib. However, p.T790M was not detected in the pleural effusion analyzed 2 months later. Notably, no adverse events were observed during treatments with EGFR-TKIs. The patient died 29 months after diagnosis, which is shorter than the expected median overall survival of 36.8 months for advanced *EGFR*-mutated NSCLC [36].

## 3. Discussion

The presented case provides further insight into the biological and clinical consequences of germline *EGFR* p.V843I in the context of coexisting somatic *EGFR* alterations. Although p.V843I has been reported in several familial NSCLC cases, its pathogenic significance remains incompletely elucidated. While some studies have classified it as a variant of uncertain significance, others have reported familial clustering, co-segregation with NSCLC, and functional findings supporting a pathogenic role [20,21,22,23,24,25]. The familial aggregation observed in the present case (Figure 2) is consistent with a possible pathogenic role of this germline alteration. However, the absence of genotyping data from all affected family members precludes definitive assessment of co-segregation. Moreover, even if all family members had been genetically tested, complete segregation would not be expected because germline *EGFR* PVs are characterized by variable penetrance and intrafamilial phenotype. This is consistent with previous reports and indicates that additional genetic or environmental factors may influence lung cancer development [14,15,16,20,22].

Most reported germline *EGFR* PVs affect the TKD encoded by exons 18–21 and frequently coexist with secondary somatic *EGFR* variants. As displayed in Table 1, the spectrum of coexisting somatic alterations is highly heterogeneous, ranging from classical activating *EGFR* variants to uncommon/atypical *EGFR* variants or to the absence of an identifiable second *EGFR* variant. Few cases have been reported with coexisting somatic alterations in cancer-relevant genes other than *EGFR* (Table 1). Collectively, these observations support a model in which many germline *EGFR* PVs contribute to hereditary susceptibility and cooperate with secondary molecular events during tumorigenesis, although the oncogenic potential of individual variants is incompletely understood [15,18].

In the present case, germline *EGFR* p.V843I coexisted with an infrequent somatic *EGFR* ex19del in the C-helix, the p.S752_I759del, a combination not reported before. Moreover, the patient’s LUAD harbored a baseline *TP53* co-mutation and subsequently acquired *EGFR* and *MET* amplifications during treatment with osimertinib and afatinib rechallenge, respectively. This molecular trajectory is consistent with a model of cooperative oncogenic evolution in which germline *EGFR* PVs interact with secondary molecular events to influence tumor development, therapeutic sensitivity, and resistance [15,24,25]. The published cases summarized in Table 1 further illustrate the marked clinical heterogeneity of hereditary *EGFR*-mutant NSCLC. Accordingly, the published reports have also demonstrated highly variable responses to different generations of EGFR-TKIs, ranging from primary resistance to prolonged clinical benefit (Table 1) [23,24,25,27,28,29].

In addition to somatic *EGFR* variants, the reported coexisting molecular alterations influencing the clinical behavior of NSCLC associated with germline *EGFR* PVs include *TP53* co-mutations, amplification of other TK-receptor-encoding genes, and bypass pathway activation [11,21]. Notably, the prospective INHERIT EGFR study demonstrated that approximately 95% of lung cancers arising in germline *EGFR* carriers harbored additional somatic *EGFR* driver mutations, strongly supporting a cooperative germline–somatic model of oncogenesis [15].

Nevertheless, exceptions have been reported. Germline *EGFR* PVs such as p.V843I, p.P848L, and several familial variants described by Li et al. have been identified without an accompanying secondary *EGFR* driver mutation, consistent with the above-mentioned heterogeneous oncogenic potential of hereditary *EGFR* variants [14,17]. However, these observations should be interpreted with caution, as differences in molecular testing methodologies, corresponding sensitivities, and sequencing depth across studies raise the possibility that cooperating oncogenic alterations may inadvertently be missed in low-input materials [15].

**Figure 3:**
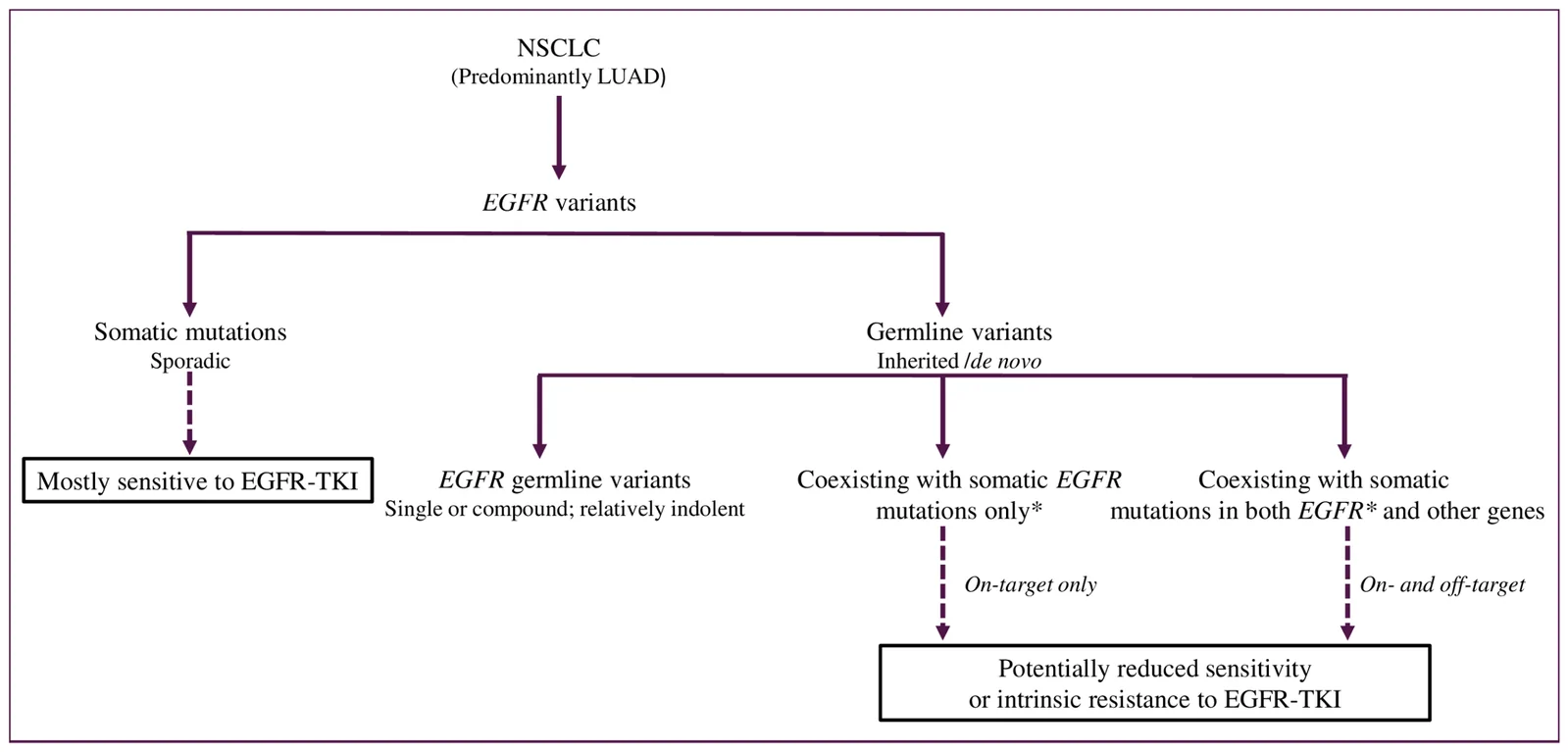
Proposed interaction between germline *EGFR* variants, secondary oncogenic alterations, and EGFR-TKI response in NSCLC. Dashed arrows: response to EGFR-TKI. Abbreviations: NSCLC: non-small-cell lung cancer. LUAD: lung adenocarcinoma. * Coexisting *in cis*.

The proposed interaction between germline and somatic *EGFR* variants or other coexisting molecular alterations, as well as the effect on therapeutic responses, is summarized in Figure 3.

To our knowledge, this is the first reported patient with coexistence *in cis* of germline *EGFR* p.V843I and somatic *EGFR* C-helix p.S752_I759del. Although p.S752_I759del is one of the infrequent ex19dels specifically occurring in the crucial regulatory αC-helix of the EGFR TKD, no significant difference in clinical characteristics and TKI-sensitivity has been observed between the C-helix ex19dels and the common ex19dels [32]. Previous reports of germline p.V843I have described this variant coexisting with somatic p.L858R or p.L861Q variants, but not with ex19dels (Table 1) [20,21,22,23,24,25,26]. Despite the presumed resistance-associated role of p.V843I, our patient initially achieved a clinically meaningful partial response to afatinib accompanied by disappearance of both the somatic *EGFR* ex19del and *TP53* variant from cfDNA. This observation may suggest that the tumor initially retained partial EGFR dependency, despite the presence of the germline variant. The temporary sensitivity to afatinib may have been influenced by the coexisting *EGFR* ex19del, a type of *EGFR* variant known to be more sensitive to afatinib than other subtypes, though treatment outcomes may vary according to the specific ex19del [37]. The p.S752_I759del variant represents an atypical ex19del subtype, and only limited clinical data regarding its sensitivity to EGFR-TKIs are available [27,28,29,31,38]. We cannot exclude that afatinib retained some activity despite the coexistence of germline p.V843I and somatic *EGFR* p.S752_I759del *in cis*, as, unlike first-generation EGFR-TKIs, afatinib irreversibly binds the EGFR kinase domain through covalent interaction with Cys797 within the ATP-binding pocket and has demonstrated inhibitory activity over germline p.T790M [26,36,38,39,40]. Whether this activity extends to germline p.V843I, particularly when it occurs *in cis* with a somatic EGFR ex19del, remains to be determined. Indeed, when germline and somatic *EGFR* variants occur *in cis*, both alterations are present within the same receptor molecule and collectively influence receptor signaling and sensitivity to EGFR-targeted therapies. Therefore, the relative contribution of the germline p.V843I variant and the infrequent ex19del to treatment response cannot be fully disentangled.

Nevertheless, the hypothesis that germline p.V843I may modify EGFR-TKI sensitivity is supported by previous clinical and experimental observations. Demierre et al. [23] reported a particularly aggressive clinical course and limited benefit from first-generation EGFR-TKIs in an NSCLC patient harboring germline p.V843I without co-existing somatic gene alterations. Moreover, Matsushima et al. [24] demonstrated in NSCLC cell lines that the combination of p.V843I and p.L858R conferred resistance to EGFR-TKIs, whereas suppression of the p.V843I allele restored drug sensitivity. Familial observations reported by Ohtsuka et al. further supported the biological and clinical relevance of this germline variant as a genetic alteration predisposing to familial NSCLC and associated with lack of objective response to EGFR-TKIs [25]. Conversely, the response to osimertinib reported by Song et al. [28] indicates that the effect of p.V843I may not be absolute and may depend on the type of accompanying somatic *EGFR* alteration and broader molecular context.

The *in cis* configuration places both the activating ex19del and the germline p.V843I substitution within the same EGFR molecule, thereby providing a biologically plausible mechanism by which p.V843I could modify receptor signaling and reduce sensitivity to EGFR-targeted therapy. In this respect, previous functional studies in vitro have shown that the p.V843I variant not only contributes to tumorigenesis by promoting phosphorylation of EGFR and its downstream signaling proteins, but similarly to the p.T790M variant, it may also provide resistance to first-generation reversible EGFR-TKIs through a structural modification that sterically hinders the drug binding to the EGFR TKD [24,33]. Both variants occur within or near the hydrophobic core and ATP-binding pocket of the TKD (exon 21 for p.V843I and exon 20 for p.T790M) and thereby result in decreased binding affinity and resistance to erlotinib and gefitinib [24,33]. Yet, the effect of p.V843I on the binding of afatinib or osimertinib to the EGFR TKD has not been fully elucidated.

Indeed, progression on afatinib occurred after only 6 months. Despite repeated tissue and liquid biopsies at progression on afatinib, no established resistance mechanism, such as histologic transformation, acquired on-target *EGFR* variants, or bypass pathway alterations, was identified. Considering the above-mentioned previous clinical and functional observations, our finding supports the possibility that germline p.V843I may have contributed to intrinsic or early adaptive resistance rather than serving exclusively as a predisposing event. However, its relative contribution cannot be distinguished from that of the coexisting somatic *EGFR* exon 19 deletion and baseline *TP53* alteration. Given the structural similarities between p.V843I and p.T790M, osimertinib was considered a rational subsequent treatment strategy. However, no clinically meaningful response was observed. Published experience with osimertinib in patients harboring germline *EGFR* PVs remains limited and is predominantly restricted to cases with p.T790M. Clinical outcomes appear heterogeneous, with both clinically meaningful responses and primary resistance reported depending on the specific germline variant and accompanying molecular alterations (Table 1) [2,25,26,27,28,29].

In this context, the lack of durable benefit from afatinib and osimertinib in our patient may not solely reflect the presence of germline p.V843I, but also the coexistence of additional molecular alterations, including a *TP53* mutation. Indeed, at baseline, the tumor also harbored the *TP53* p.E258K co-mutation, which may have contributed to adverse tumor biology and represents an alternative or complementary explanation for the limited duration of response. *TP53* co-mutations are common in sporadic *EGFR*-mutated NSCLC and have consistently been associated with shorter progression-free survival during EGFR-TKI treatment [35,38,41,42,43,44,45]. However, their prevalence and prognostic significance in hereditary *EGFR*-associated NSCLC remain unknown because the available evidence is limited to individual cases and small series. The specific *TP53* p.E258K alteration is a hotspot missense variant located within the p53 DNA-binding domain. As for other *TP53* mutations within this domain, p.E258K has been verified both in vitro and in vivo as a loss-of-function mutation resulting in loss of tumor-suppressive capabilities of p53 as a transcriptional regulator of cell cycle, DNA repair, and apoptosis [41,46,47]. Nevertheless, despite available functional evidence supporting impaired p53 activity by the p.E258K variant, its independent effect on EGFR-TKI sensitivity has not yet been established by variant-specific clinical data.

In contrast, *EGFR* amplification and subsequent *MET* amplification were not present at baseline but emerged later during fourth-line treatment with osimertinib and fifth line as afatinib-rechallenge, respectively. These alterations therefore cannot explain the relatively short duration of benefit from third-line afatinib and should instead be interpreted as later acquired resistance events reflecting temporal clonal evolution under therapeutic selective pressure. Although *TP53* dysfunction may promote genomic instability and clonal diversification, a direct causal relationship between *TP53* p.E258K and the later emergence of *EGFR* or *MET* amplification cannot be established from this individual case. The transient detection of somatic *EGFR* p.T790M in cfDNA, followed by its absence in pleural fluid, further supports substantial spatial and temporal molecular heterogeneity.

From a clinical perspective, identification of a germline *EGFR* PV has implications beyond therapeutic decision-making. Detection of an apparently pathogenic *EGFR* variant, particularly in younger never-smokers with NSCLC, and unusual treatment responses, should prompt confirmation of germline status using non-tumor DNA, careful assessment of family history, and consideration of referral for genetic counseling. Clinicians should also be aware that coexisting germline *EGFR* PVs may modify treatment sensitivity and resistance patterns. This supports the use of comprehensive longitudinal molecular profiling throughout the disease course to guide appropriate management of patients with clinically relevant germline alterations. Indeed, as illustrated by the present case, longitudinal monitoring of patients by analyzing serial tissue and liquid biopsies allows for the assessment of tumor heterogeneity, clonal evolution, and resistance dynamics that may characterize NSCLC with germline *EGFR* variants (Figure 1) [13,15,27,29,31]. As comprehensive NGS becomes increasingly implemented in routine clinical practice, incidental identification of germline *EGFR* PVs is expected to become more frequent, emphasizing the need for multidisciplinary interpretation of these findings involving clinical geneticists.

The lack of benefit from immunotherapy observed in our patient is also consistent with accumulating evidence demonstrating limited efficacy of immune checkpoint inhibitor monotherapy in *EGFR*-mutated NSCLC, which has been attributed in part to the distinct immune microenvironment and lower immunogenicity of *EGFR*-driven tumors [40,48].

Several limitations should be acknowledged. This report describes a single patient and therefore cannot establish a causal relationship between germline p.V843I and reduced sensitivity to EGFR-TKIs. Moreover, multiple molecular events, including the *TP53* variant coexisting at baseline and the *EGFR* amplification and *MET* amplification acquired during EGFR-TKI therapy, may also have contributed to treatment resistance and tumor evolution. Additional clinical cases and confirmatory functional studies in cellular and/or mouse models would be required to confirm the oncogenic interactions of somatic ex19del (p.S752_I759del) and germline p.V843I and their combined impact on sensitivity to different generations of EGFR-TKIs.

**Table 1:** Germline variants in *EGFR* exons 19, 20 and 21 coexisting with somatic alterations. Coexisting somatic variants were not always specified in the cited references. Abbreviations: LUAD: lung adenocarcinoma. LUSC: lung squamous carcinoma. ASC: adenosquamous carcinoma. NR: not reported. PFS: progression-free survival.

Germline Variant	Variant Position	Coexisting Somatic *EGFR* Mutations/Other Mutations ^§^	Tumor Histology	EGFR-TKI Treatment	Reported PFS	Reference
***EGFR*** ** exon 19**	**Ex19dels**	p.S768I, p.L788V, p.T790M, p.L858R	LUAD	NR	NR	[14]
	**p.K757R**	Ex19del	LUAD	icotinib osimertinib	36 months 12 months	[19]
***EGFR*** **exon 20**	**p.V769M**	Ex19del, p.G719A, p.L858R, *EGFR* amp, *MET* amp	LUAD	NR	NR	[49]
		p.G719X	LUAD	erlotinib	>36 months	[50]
	**p.V769indelsVASV**	p.S768N, p.L858R	LUAD	NR	NR	[14]
	**p.G719D**	p.L861R	LUAD	NR	NR	[49]
	**p.G724S**	p.S768I	LUAD	gefitinib	1 month	[19]
	**p.S768I**	p.Q791 *	LUAD	NR	NR	[14]
	**p.R776G**	p.L858R	LUAD	NR	NR	[51]
	**p.R776H**	p.G719A	LUSC	NR	NR	[18]
		p.G719S	LUSC	erlotinib	>12 months	[18]
		p.G719S, p.L861Q	LUAD	almonertinib	>18 months	[16]
		p.L858R	LUAD	NR	NR	[49]
		p.L861Q	LUAD	gefitinib osimertinib	23 months	[52]
	**p.V786M**	p.L858R	LUAD	gefitinib	3 months	[19]
	**p.T790M**	Not specified	LUAD	NR	NR	[6,9]
		Ex19del Ex19del, *TP53* p.R213 *, *PIK3CA* p.E545K	LUAD LUAD ASC LUAD LUAD	Unspecified NR afatinib osimertinib gefitinib osimertinib	NR NR 12 months NR 9 months 19 months	[8,49,53,54,55]
		Ex19del, p.G719A, p.L858R	LUAD	NR	NR	[6]
		Ex19del, p.G719S, p.L858R	LUAD	erlotinib	1 month to >5 years (several probands)	[56]
		Ex19del, p.L858R, *ARIDIA* p.K1938N	LUAD	erlotinib	NR	[13]
		p.G719A	LUAD	osimertinib	>5 months	[57]
		p.G719R	LUAD	afatinib osimertinib	NR	[53]
		p.G719S	LUAD LUAD	NR NR	NR NR	[19,58]
		p.L858R	LUAD LUAD ASC LUAD	NR NR osimertinib icotinib osimertinib	NR >48 months >30 months 3 months 15 months	[7,8,27,29]
		p.L858R, p.L861Q, *EGFR* amp, ^¶^ *MYC* amp, ^¶^ *RICTOR* amp, ^¶^ Mutation of *CPEB3, FAS*, *FLT3, SLC34A2,* *SMARCA4*	LUAD LUAD LUAD	NR NR NR	NR NR NR	[14,49,59]
	**p.L792F**	Ex19del	LUAD	NR	NR	[19]
***EGFR*** **exon 21**	**p.R831H**	p.L858R	LUAD	erlotinib	10 months	[19]
		KRAS	LUAD	almonertinib	20 days	[60]
	**p.V843I**	Ex19del (infrequent C-helix ex19del)	LUAD	afatinib osimertinib	6 months 1.5 months	Current case
		p.L858R	LUAD LUAD LUAD	erlotinib gefitinib erlotinib NR	NR NR NR	[17,22,26]
		p.L858R, p.L861Q	LUAD	None	-	[21]
		p.L858R, *TP53* p.R248W	LUAD	erlotinib, gefitinib	NR	[25]
	**p.V834L**	p.L858R	LUAD	erlotinib osimertinib	12 months	[61]
	**p.L844V**	Not specified	LUAD	icotinib afatinib	3 months 13 months	[19]
	**p.P848L**	Ex19del	LUAD	erlotinib	NR	[62]
	**p.A871E**	p.L858R	LUAD	osimertinib	18 months	[63]
	**p.V1010M**	p.L858R	LUAD	gefitinib afatinib	4 months 3 months	[64]
	**p.D1014N**	p.L858R	LUAD	gefitinib	29 months	[19]

^§^ May include 1 or more probands. * Indicates a nonsense mutation in the gene that results in a premature stop codon and truncation of the corresponding protein. ^¶^ These somatic gene alterations were only reported in [59], as detected by whole-exome sequencing. amp: amplification of the indicated gene.

## 4. Materials and Methods

### 4.1. Immunohistochemistry (IHC)

IHC was performed on FFPE 2.5 μm thick tissue sections using a BenchMark ULTRA automated slide immunostainer (Ventana Medical Systems, Inc., Roche Diagnostics; Hvidovre, Denmark). The pre-diluted Ventana rabbit mAbs D5F3 against ALK, SP384 against ROS-1, and CONFIRM SP44 anti-Total c-MET (Ventana, Roche Diagnostics; Hvidovre, Denmark) were employed according to the manufacturer’s instructions and staining conditions, including the usage of the corresponding negative control (sections stained with an unrelated matched rabbit IgG mAb) and the positive control, as previously described [35]. Programmed cell death ligand 1 (PD-L1) expression was assessed by immunohistochemistry in the PD-L1 IHC 22C3 pharmDx kit (Agilent-Dako, Glostrup, Denmark) on the Dako ASL48 platform.

### 4.2. Fluorescence in Situ Hybridization (FISH)

The 2 μm thick FFPE tissue sections from biopsies were hybridized overnight with the Zyto-Light SPEC MET/CEN7 Dual Color Probe (ZytoVision, Bremerhaven, Germany) that detects the *MET* gene and the centromeric portion of the *MET*-harboring chromosome 7, as previously described [35].

### 4.3. Mutation Analysis

The diagnosis of the pulmonary adenocarcinoma was initially tested for *EGFR* variants using the Cobas^®^ *EGFR* Mutation Test v2 (Roche Diagnostics) on the Cobas z480 analyzer (analytical sensitivity of 5%) according to the supplier’s instructions. This fully automated analysis covered 42 *EGFR* variants, including p.G719X (p.G719A, p.G719C, or p.G719S) in exon 18, 29 most common ex19dels, p.S768I and p.T790M in exon 20, five different exon 20 insertions, and p.L858R (two distinct nucleotide variants) as well as p.L861Q in exon 21.

To identify TKI-resistance mechanisms during treatment, the baseline biopsy and the three longitudinal re-biopsies from new consecutive metastatic lesions emerging during the disease evolution were analyzed histologically and by IHC, FISH, and targeted next-generation sequencing (NGS) on DNA and RNA. For targeted NGS of DNA hot-spot variants in 22 genes relevant for NSCLC, 10 ng of genomic DNA were purified from each FFPE specimen using the QIAamp DNA Minikit (Qiagen AB, Vedbæk, Denmark), quantified by the Qubit^®^ dsDNA HS assay (ThermoFisher Scientific, Roskilde, Denmark), and subsequently analyzed using the Ion AmpliSeq Colon-Lung Cancer Research Panel v2 (ThermoFisher Scientific), as previously described [35]. After preparation of amplicon-based libraries, sequencing was performed on an Ion Torrent Personal Genome™ (PGM) sequencer (Thermo Fisher Scientific) using the Ion PGM Sequencing 200 Kit v2 according to the manufacturer’s instructions. Data analysis, including alignment to the hg19 human reference genome and variant calling, was performed by the Torrent Suite Software v.4.4 (ThermoFisher Scientific) and visually verified with the Integrative Genomics Viewer (IGV) v.2.1 (Broad Institute, Burlington, MA, USA).

As not all possible *EGFR* variants were covered by this targeted NGS panel, direct Sanger dideoxy-sequencing of the *EGFR* gene’s TKD (exon 18–21) was performed on DNA from circulating lymphocytes and from the diagnostic biopsy as previously reported [65].

Further assessment of the tumor biopsies with broader coverage of hot-spot variants such as relevant SNVs, indels and CNVs across 52 genes was performed using the Oncomine Focus Assay (ThermoFisher Scientific), as previously reported [66]. In this case, the DNA was sequenced on the Ion Torrent™ GeneStudio™ S5 Plus System (ThermoFisher Scientific) according to the manufacturer’s instructions. Additionally, liquid biopsies (cfDNA from plasma) were analyzed for relevant DNA variants by the Oncomine Lung cfDNA NGS assay according to the manufacturer’s instructions (ThermoFisher Scientific).

RNA isolated from the biopsies was analyzed by multiplex PCR/NGS assay for gene fusions utilizing the Archer^®^ anchored multiplex PCR (AMP™)/NGS assay (FusionPlex^®^ Solid Tumor Kit) according to the manufacturer’s instructions (ArcherDX, Inc., Boulder, CO, USA), as previously described [66].

### 4.4. Ethical Aspects

The patient’s family gave informed written consent for the publication of this paper, including clinical data and images of the deceased patient. Moreover, the patient had not previously registered a denial of the usage of her own tissue for research purposes in the Danish Registry for Tissue Usage. The study was conducted in accordance with the Declaration of Helsinki and the ethical guidelines of the Capital Region of Denmark. The case was part of a clinical study for NSCLC patients at our institution that was approved by the local ethical committee of Rigshospitalet, Copenhagen University Hospital and by the Danish Capital Region’s Committee for Health Research Ethics (project identification code and approval number: H-15008619).

## 5. Conclusions

To our knowledge, this is the first reported case of advanced LUAD with germline *EGFR* p.V843I coexisting *in cis* with an infrequent somatic *EGFR* ex19del and a somatic *TP53* variant. The case was characterized by a transient and short-lived response to afatinib, lack of clinical benefit from osimertinib, and dynamic molecular evolution documented through longitudinal analysis of serial tissue and liquid biopsies. Our findings, considered together with previous clinical reports and functional studies involving germline p.V843I combined with different somatic *EGFR* mutations, support the hypothesis that this germline variant may not only function as a tumor-predisposing event but may also modify the sensitivity to EGFR-TKIs. However, as in previous cases harboring other somatic and germline *EGFR* mutant configurations, the independent contribution of germline p.V843I cannot be established in the present case because of the coexisting somatic *EGFR* exon 19 deletion and baseline *TP53* variant. Collectively, these findings highlight hereditary *EGFR*-mutated NSCLC as a clinically and biologically heterogeneous disease posing significant diagnostic and therapeutic challenges for oncologists, pathologists, and clinical geneticists.

## Data Availability

The data presented in this study are available upon request from the corresponding author. The data are not publicly available because of Danish data protection regulations (GDPR) and ethical restrictions. .

## Abbreviations

The following abbreviations are used in this manuscript:

cfDNA	Circulating free DNA
Ex19del	Exon 19 deletion
FFPE	Formalin-fixed, paraffin-embedded
IHC	Immunohistochemistry
LUAD	Lung adenocarcinoma
NSCLC	Non-small-cell lung cancer
NGS	Next-generation sequencing
PV	Pathogenic variant
RECIST	Response evaluation criteria in solid tumors
TKD	Tyrosine kinase domain
TKI	Tyrosine kinase inhibitor
